# Down-regulation of IGHG1 enhances Protoporphyrin IX accumulation and inhibits hemin biosynthesis in colorectal cancer by suppressing the MEK-FECH axis

**DOI:** 10.1515/biol-2021-0098

**Published:** 2021-09-06

**Authors:** Guangjian Yang, Gang Li, Xuemei Du, Wenting Zhou, Xiaohong Zou, Yuanfu Liu, Hong Lv, Zhenjiang Li

**Affiliations:** Department of Pathology, The First People’s Hospital of Longquanyi District of Chengdu, Chengdu, Sichuan, 610100, China; Department of Anorectal, The First People’s Hospital of Longquanyi District of Chengdu, No. 201, Group 3, Chengdu, Sichuan, 610100, China; Department of Research and Development, Sichuan Haosidelifu Science and Technology Ltd, Chengdu, Sichuan, 610041, China

**Keywords:** IGHG1, protoporphyrin IX, hemin, colorectal cancer, MEK-FECH

## Abstract

Immunoglobulin γ-1 heavy chain constant region (IGHG1) is a functional isoform of immunoglobulins and plays an important role in the cytolytic activity of immune effector cells. Dysregulated IGHG1 was implicated in the occurrence and development of various tumors. Protoporphyrin IX (PpIX) is an endogenous fluorophore and is used in photodynamic therapy, which induces the generation of reactive oxygen species to initiate the death of tumor cells. However, the roles of IGHG1 in the colorectal cancer cell proliferation and PpIX accumulation have not been reported yet. Data from qRT-PCR and western blot analysis showed that IGHG1 was up-regulated in the colorectal cancer cells. Colorectal cancer cells were then transfected with shRNA targeting IGHG1 to down-regulate IGHG1 and conducted with Cell Counting Kit 8 (CCK8) and colony formation assays. Results demonstrated that shRNA-mediated down-regulation of IGHG1 decreased cell viability of colorectal cancer and suppressed cell proliferation. Moreover, PpIX accumulation was promoted and the hemin content was decreased by the silence of IGHG1. Interference of IGHG1 reduced the phosphorylated extracellular signal-regulated kinase (ERK) and ferrochelatase (FECH) expression, resulting in retarded cell proliferation in an MEK-FECH axis-dependent pathway.

## 1 Introduction

Colorectal cancer is one of the most common tumors globally, with a death rate of 13.7/year per 100,000 persons [1,2]. The 5-year overall survival rate of patients with colorectal cancer at the early stage is about 90%, while approximately 30% for the patients who suffer lymph nodes or distant nodes metastases [3]. Therefore, the diagnosis of colorectal cancer is important for the treatment of cancer. Nowadays, surgery, radiotherapy, and chemotherapy are the major therapeutic strategies for colorectal cancer, while the recurrence rate of colorectal cancer is high in patients post-surgery [4]. Patients with advanced colorectal cancer often face the risk of tumor metastasis, so novel strategies are required to prolong the survival rate of the patients [5].

Fluorescence-guided surgery with exogenous administration of 5-aminolevulinic acid has been used as the preoperative imaging technique for cancer detection [6]. 5-Aminolevulinic acid is metabolically converted to protoporphyrin IX (PpIX), which functions as an endogenous fluorophore and is used in fluorescence-guided surgery for the detection of colorectal cancer [7]. Moreover, PpIX has been found to be accumulated in the cancer cells and can increase the sensitivity of radiotherapy [8]. PpIX, with the ability to induce DNA double-strand break and reduce damage repair, was commonly used in photodynamic therapy [9]. PpIX is complexed with Fe^2+^ to produce heme under the catalysis of ferrochelatase (FECH), and heme was reported to be related to the malignant process of colorectal cancer [10]. Therefore, the promotion of PpIX accumulation and the inhibition of heme biosynthesis might inhibit colorectal cancer progression and provide ideas for increasing the sensitivity of radiotherapy and photodynamic therapy.

As a functional isoform of immunoglobulins, immunoglobulin γ-1 heavy chain constant region (IGHG1) is implicated in the cytolytic activity of immune effector cells [11]. Enhanced expression of IGHG1 is closely associated with the occurrence of breast cancer [12]. IGHG1 has been shown to promote the metastasis of gastric [13] and ovarian [14] cancer. Inhibition of IGHG1 suppressed the cell proliferation of prostate cancer and promoted cell apoptosis [15]. In addition, inhibition of IGHG1 suppressed prostate cancer growth through inactivation of MEK/ERK pathway [16], and suppression of MEK activation promoted the accumulation of PPIX in colon cancer cells [17]. IGHG1 was hypothesized to regulate PpIX accumulation and heme biosynthesis during the development of colorectal cancer in this study.

## 2 Materials and methods

### 2.1 TCGA analysis

GEPIA database (http://gepia.cancer-pku.cn/) was used to plot the gene expression level of IGHG1 between 275 colon adenocarcinoma tissues and 349 normal tissues in TCGA RNA-seq raw data.

### 2.2 Cell culture and transfection

Human intestinal epithelial cells (HIEC) and colorectal cancer cells (HT29, SW480, SW620, HCT116) were obtained from the American Type Culture Collection (Manassas, VA, USA). Cells were cultured in DMEM medium (30-2002; Wisent Corporation, Wisent, Canada) containing 10% fetal bovine serum (502; Wisent Corporation) in a humidified incubator at 37°C. HT29 cells were seeded in a 96-well plate for 24 h to achieve 80% confluence and then transfected with shRNA targeting IGHG1 (Genepharma, Shanghai, China) by Lipofectamine 2000 (11668019; Sigma Aldrich, St. Louis, MO, USA).

### 2.3 qRT-PCR

HIEC, HT29, SW480, SW620, and HCT116 cells were incubated with Trizol (R0016; Takara, Shiga, Japan) for RNA isolation. The RNAs were reverse-transcribed into cDNAs using PrimeScript™ RT reagent kit (RR037B; TaKaRa). qRT-PCR analysis of IGHG1 was performed using SYBR Green Mix (RR091B; Takara) with the following conditions: 95°C for 10 min, 40 cycles of 95°C for 15 s and 60°C for 1 min. GAPDH was used as the endogenous control, and the specific primers were indicated as follows: GAPDH forward: 5′-GAAGGTGAAGGTCGGAGT-3′ and reverse: 5′-GAAGATGGTGATGGGATTTC-3′; and IGHG1 forward: 5′-ACTCCGACGGCTCCTTCTTC-3′ and reverse: 5′-TTCTGCGTGTAGTGGTTGTGC-3′.

### 2.4 Cell viability and proliferation

HT29 cells were seeded into the 96-well plate for 24, 48, or 72 h. CCK8 solution (C0037; Beyotime, Beijing, China) was added to each well and incubated for 2 h. Absorbance at 450 nm was measured using a microplate reader (Bio-Rad, Hercules, CA, USA) to detect the cell viability. For cell proliferation assay, HT29 cells were seeded in the 6-well plate and cultured for 14 days. Paraformaldehyde-fixed and crystal violet-stained cells were observed under a light microscope (Olympus, Tokyo, Japan).

### 2.5 Flow cytometry

#### 2.5.1 PpIX and heme measurement

Cellular fluorescence of PpIX in HT29 was measured under FACS Calibur (BD Biosciences, San Jose, CA, USA) with excitation wavelength at 488 nm and emission wavelength at 650 nm. Data were analyzed using FlowJo (LLC, Ashland, OR, USA). HT29 cells were homogenized in the hemin assay buffer from Hemin assay kit (MAK036; Sigma Aldrich). The cell lysates were incubated with hemin substrate, hemin probe, and enzyme mix for 1 h. Absorbance at 570 nm was detected using a Synergy MX plate reader (BioTek Instruments Inc. Winooski, VT, USA) for the determination of heme content.

### 2.6 Ferrochelatase activity

The lysate of HT29 cells was incubated with 200 µM PpIX (Sigma Aldrich) in assay buffer (0.3% v/v Tween 20 and 1 mM palmitic acid in 0.1 M tris-HCl, pH 8.0). Zinc acetate solution (50 µL; 2 mM) was added into the cell lysate and incubated for 2 h. Ice-cold stop buffer (1 mM EDTA in 30:70 DMSO/methanol) was used to terminate the reaction. Following centrifugation in 14,000 g for 10 min, the intensity of fluorescence at 590 nm (with 405 nm excitation wavelength) was measured under the Synergy MX plate reader to determine the formation of zinc-protoporphyrin IX.

### 2.7 Western blot

HT29 cells were lysed in RIPA buffer (WB-0071; Ding Guo Chang Sheng Biotech, Beijing, China), and centrifugated at 12,000 g for 1 h to harvest the supernatant. The protein concentration was calculated using the bicinchoninic acid protein assay kit (BCA1-1KT; Sigma Aldrich). Samples (40 µg) were separated using SDS-PAGE and then transferred to the PVDF membrane. The membranes were blocked with 5% non-fat milk and then probed with anti-IGHG1 (1:2,000; ab108969; Abcam, Cambridge, MA, USA), anti-ERK (ab54230) and anti-p-ERK (1:2,500; ab192591; Abcam), anti-FECH (1:3,000; ab219349; Abcam), and anti-β-actin (1:3,500; ab8227; Abcam). Following incubation with horseradish peroxidase-conjugated immunoglobulin G (1:4,000; ab6721; Abcam), the blots were detected using enhanced chemiluminescence (KeyGen, Nanjin, China).

### 2.8 Statistical analysis

Results from three independent experiments were presented as mean ± SD. Statistical analyses between different groups were performed with one-way analysis of variance or Student’s *t*-test using SPSS 19.0 software (Armonk, NY, USA). *p* < 0.05 was considered as a significant difference.

## 3 Results

### 3.1 Up-regulation of IGHG1 in colorectal cancer

**Figure 1:**
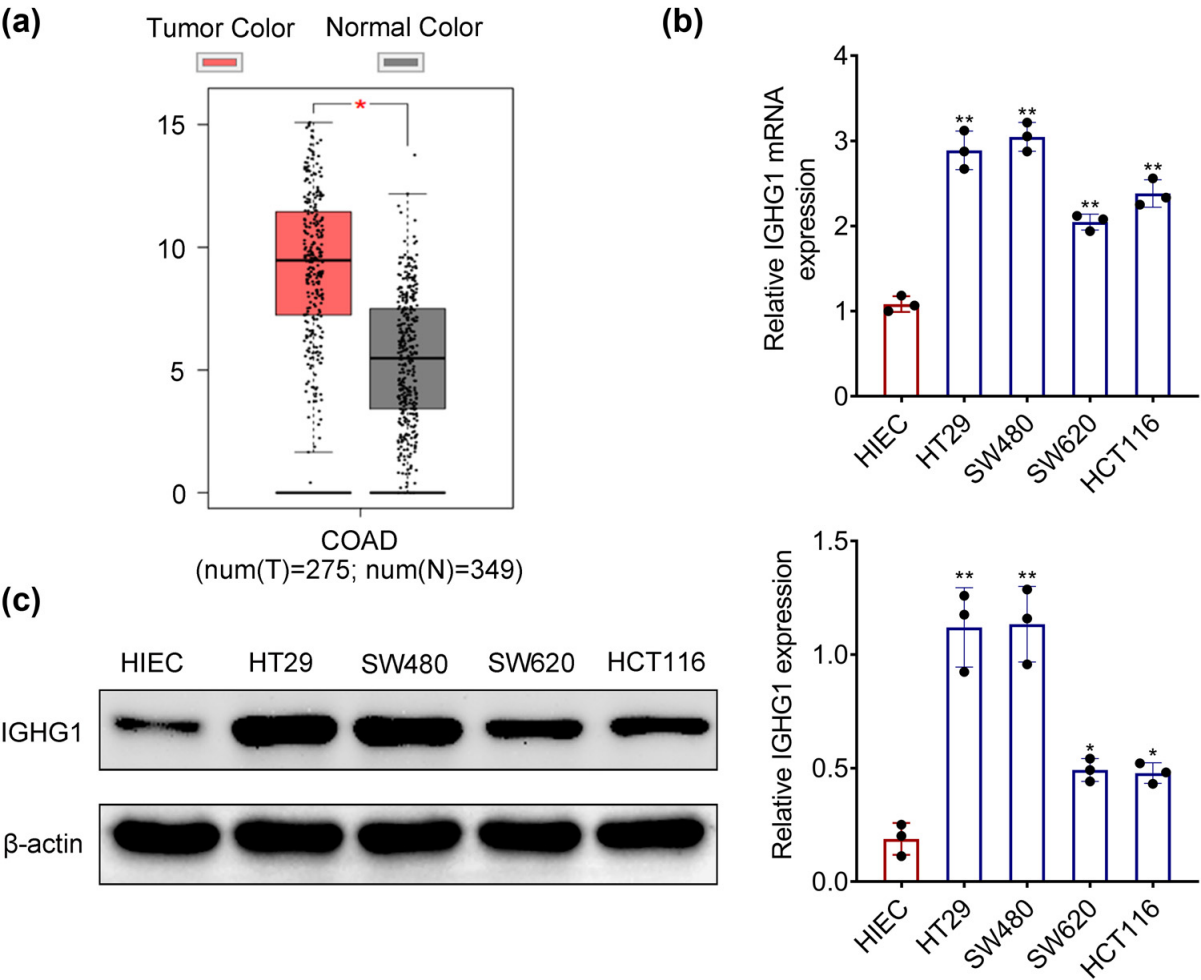
Up-regulation of IGHG1 in colorectal cancer. (a) IGHG1 was highly expressed in colorectal cancer tissues compared to the normal tissues based on TCGA database. (b) IGHG1 was highly expressed in colorectal cancer cells (HT29, SW480, SW620, HCT116) compared to human intestinal epithelial cells (HIEC) based on qRT-PCR analysis. (c) IGHG1 was highly expressed in colorectal cancer cells (HT29, SW480, SW620, HCT116) compared to human intestinal epithelial cells (HIEC) based on western blot analysis. *, ** vs HIEC, *p* < 0.05, *p* < 0.01.

Higher expression of IGHG1 was identified in the colorectal cancer cells (HT29, SW480, SW620, and HCT116) compared to human intestinal epithelial cell (HIEC) (Figure 1b and c), suggesting that IGHG1 might be implicated in colorectal cancer progression.

### 3.2 IGHG1 contributed to colorectal cancer cell proliferation

**Figure 2:**
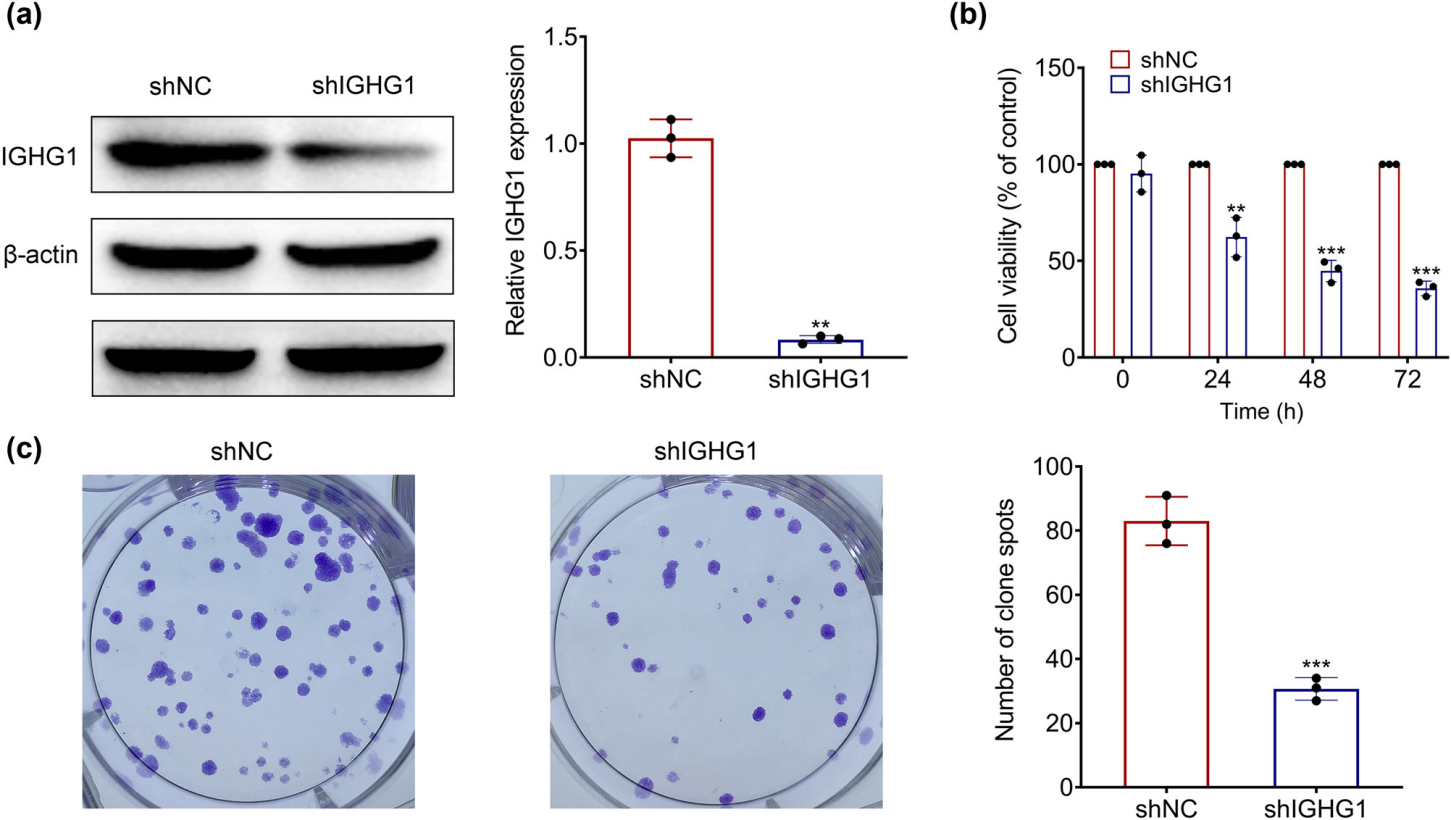
IGHG1 contributed to colorectal cancer cell proliferation. (a) The expression of IGHG1 in HT29 cells transfected with shIGHG1 and shNC. (b) Knockdown of IGHG1 decreased cell viability of HT29cells. (c) Knockdown of IGHG1 repressed cell proliferation of HT29 cells. **, *** vs shNC, *p* < 0.01, *p* < 0.001.

HT29 was transfected with shRNA targeting IGHG1 to investigate the functional role of IGHG1 in colorectal cancer. IGHG1 was down-regulated in HT29 cells transfected with shIGHG1 (Figure 2a). Knockdown of IGHG1 inhibited the cell viability and proliferation of HT29 cells (Figure 2b and c), demonstrating that silence of IGHG1 had an anti-proliferative effect on colorectal cancer cells.

### 3.3 IGHG1 mediated PpIX accumulation and heme biosynthesis in colorectal cancer

**Figure 3:**
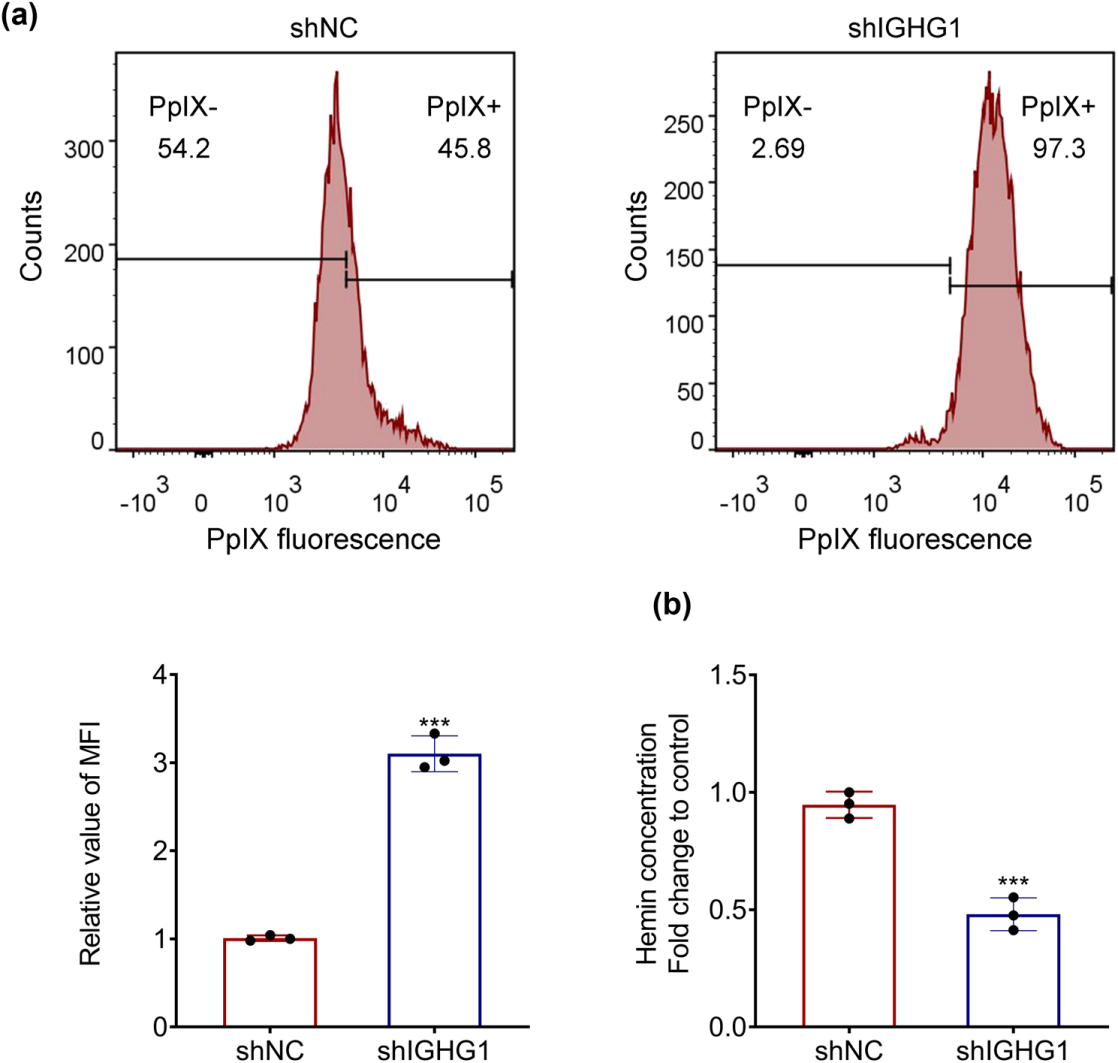
IGHG1 mediated PpIX accumulation and heme biosynthesis in colorectal cancer. (a) Knockdown of IGHG1 enhanced cellular fluorescence of PpIX in HT29 cells. (b) Knockdown of IGHG1 reduced heme content in HT29 cells. *** vs shNC, *p* < 0.001.

To determine the effect of IGHG1 on PpIX accumulation, HT29 cells transfected with shIGHG1 and shNC (negative control group) were conducted with flow cytometry analysis. Compared with control, cells transfected with shIGHG1 showed higher intensity of cellular fluorescence of PpIX (Figure 3a), indicating that knockdown of IGHG1 enhanced PpIX accumulation in the colorectal cancer cell. Moreover, heme content in HT29 cells was reduced by knockdown of IGHG1 (Figure 3b), revealing that knockdown of IGHG1 suppressed heme biosynthesis in the colorectal cancer cell.

### 3.4 IGHG1 mediated MEK-FECH signaling activation in colorectal cancer

**Figure 4:**
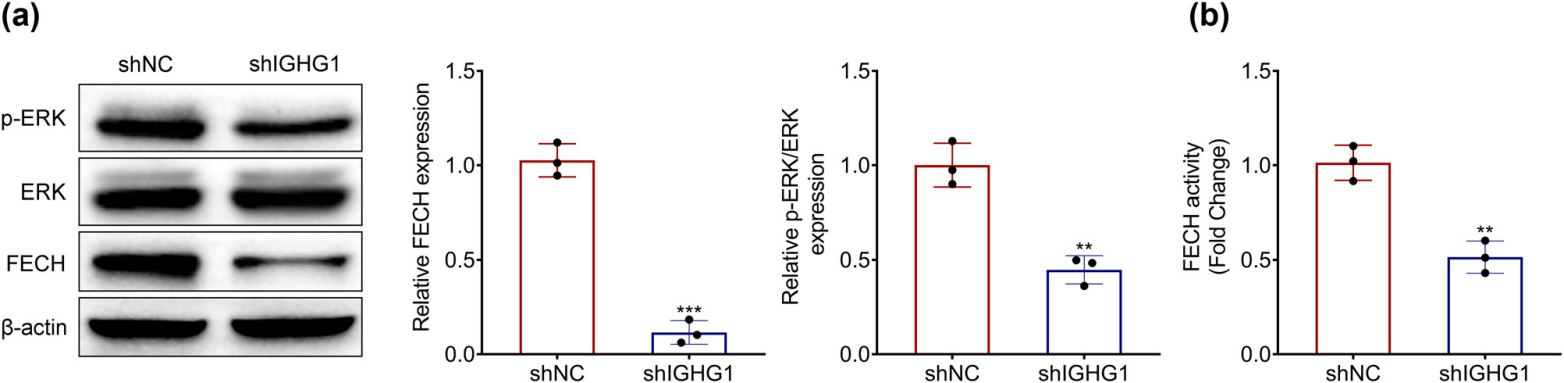
IGHG1 mediated MEK-FECH signaling pathway in colorectal cancer. (a) Knockdown of IGHG1 reduced expression of phosphorylated ERK and FECH in HT29 cells. (b) Knockdown of IGHG1 reduced FECH activity in HT29 cells. **, *** vs shNC, *p* < 0.01, *p* < 0.001.

Knockdown of IGHG1 reduced the expression and the activity of FECH protein, an enzyme involved in heme biosynthesis, in HT29 cells (Figure 4a and b), which indicated that IGHG1 contributed to FECH activation in colorectal cancer. In addition, phosphorylated ERK was also decreased by knockdown of IGHG1 in HT29 cells (Figure 4a), showing that silence of IGHG1 retarded MEK-FECH signaling transduction in colorectal cancer.

## 4 Discussion

Immunoglobulins are aberrantly expressed in the tumor cells [18]. High level of immunoglobulin M and low level of immunoglobulin A function as a prognostic indicator of colorectal cancer [19]. Cancer immunoglobulin G was also dysregulated in colorectal cancer [20] and participated in colorectal cancer progression [21]. Considering IGHG1 was involved in the tumorigenesis of a variety of cancers, the functional role of IGHG1 in colorectal cancer was investigated in this study.

IGHG1 was found to be enhanced in the colorectal cancer cells. Moreover, data based on TCGA (http://gepia.cancer-pku.cn/) analysis showed that the expression of IGHG1 was also elevated in the colorectal cancer tissues (Figure 1a). Therefore, IGHG1 might be involved in colorectal cancer progression. Knockdown of IGHG1 decreased the cell viability of colorectal cancer and suppressed cell proliferation. These results suggested that IGHG1 contributed to colorectal cancer cell proliferation.

Heme biosynthesis exists in all types of cells and plays a crucial role in cell metabolism, including oxygen transport, cell oxidation regulation, and drug metabolism [22]. In addition, heme is implicated in the pathogenesis of various diseases, such as tumors [23]. Heme accumulation has been reported to induce cytotoxic heme factor formation, which results in cytotoxic damage to colonic epithelial cells [23]. Moreover, heme also alters the intestinal flora to enhance heme-induced cytotoxic effects, thus leading to colorectal carcinogenesis [23]. Here, the silence of IGHG1 reduced the heme content in HT29 cells and exerted anti-tumor role. Additionally, PpIX, the intermediate in heme biosynthesis pathway, has been shown to induce the production of reactive oxygen species under visible light excitation, thus leading to the death of cancer cells [24]. Inducing accumulation of intracellular PpIX has been reported to be a small-molecule-based approach for treating colon cancer [25]. In the present study, cellular fluorescence of PpIX was enhanced by knockdown of IGHG1 in the colorectal cancer cells, indicating that IGHG1 might be a potential therapeutic target for the treatment of colorectal cancer through regulation of PpIX accumulation and heme biosynthesis.

MEK pathway is activated by inducing mutations of major oncogenic proteins or growth factors, thus leading to enhanced cellular growth, invasion, development, and resistance to therapy during carcinogenesis and maintenance of colorectal cancer [26]. Inactivation of oncogenic MEK/ERK pathway promoted the suppression of colorectal cancer progression [27]. Moreover, activation of oncogenic Ras/MEK pathway promoted the activity of FECH, thus reducing the PpIX accumulation [28]. The repression of oncogenic Ras/MEK signaling pathway reduced the activity of FECH and increased PpIX accumulation, which blocked breast cancer progression both *in vitro* and *in vivo* [17]. This study showed that knockdown of IGHG1 reduced the phosphorylation of extracellular signal-regulated kinase (ERK) and reduced expression (and therefore activity) of ferrochelatase (FECH) in colorectal cancer cells, revealing that IGHG1 silence might repress the progression of colorectal cancer through inactivating the MEK-FECH signaling pathway.

In summary, IGHG1, up-regulated in the colorectal cancer tissues and cells, contributed to the proliferation of colorectal cancer cells through increasing heme biosynthesis and decreasing PpIX accumulation. In the MEK-FECH axis-dependent pathway, suggesting that IGHG1 might be a novel potential therapeutic target for colorectal cancer treatment. However, the *in-vivo* regulatory role of IGHG1 in colorectal cancer should be further investigated to confirm the anti-tumor effect of IGHG1 silence. Moreover, the effect of IGHG1 on colorectal cancer cell migration and invasion will be investigated in further research.
